# COVID-19 and Diabetic Ketoacidosis: A Single Center Experience

**DOI:** 10.7759/cureus.13000

**Published:** 2021-01-30

**Authors:** Balraj Singh, Parminder Kaur, Prem Patel, Ro-Jay Reid, Abhishek Kumar, Supreet Kaur, Nirmal Guragai, Abanoub Rushdy, Mahesh Bikkina, Fayez Shamoon

**Affiliations:** 1 Hematology/Oncology, Saint Joseph's University Medical Center, Paterson, USA; 2 Cardiology, Saint Joseph's University Medical Center, Paterson, USA; 3 Internal Medicine, Saint Joseph's University Medical Center, Paterson, USA; 4 Hematology/Oncology, Jacobi Medical Center, New York, USA

**Keywords:** covid-19, severe acute respiratory syndrome-coronavirus-2, sars-cov-2 (severe acute respiratory syndrome coronavirus -2), diabetic ketoacidosis (dka), hyperglycemic hyperosmolar non-ketotic syndrome, hyperglycemic crisis, dka, type i diabetes mellitus, diabetes type 2

## Abstract

Background and objectives: To describe the clinical characteristics and outcomes of hospitalized coronavirus disease 2019 (COVID-19) patients with diabetic ketoacidosis (DKA) -- a single center tertiary hospital experience.

Materials and Methods: A retrospective study was conducted among patients admitted to our hospital in the United States between March 1^st ^and June 15^th^, 2020 with DKA and severe acute respiratory syndrome-coronavirus-2 (SARS-CoV-2) infection known as COVID-19. We compared the baseline characteristics, laboratory data, and clinical course between survivors and nonsurvivors to identify the risk factors associated with mortality in the patients with DKA.

Results: A total number of 43 patients were included in this study. The median age was 52 years. Thirty-three (76.7%) patients were male. Median value of initial glucose on presentation was 553 mg/dL (300.0-1927.0 mg/dL). On admission, 33 (76.7%) patients had glycated hemoglobin (HbA1c) ≥ 8% (64 mmol/mol) and HbA1c was not obtained in 10 (23.3%) patients. Acute kidney injury (AKI) was seen in 37 (86.0%) patients, 6 (14%) patients required renal replacement therapy and 22 (51.2%) required mechanical ventilation. Among the 43 patients, 25 (58.1%) died. Out of 25 patients who died 15 (60.0%) were Hispanics, 6 (24.0%) were White, 3 (12.0%) were African American, 1 (4%) was Arabic, and 1 (4%) was Asian. The patients who died were older in age than who survived (mean age 58 ± 6.13 vs 46 ± 9.39; p = 0.023). Some 95% of the patients requiring mechanical ventilation died (odds ratio [OR]: 89.25; 95% confidence interval [CI]: 9.10-874.96); p = 0.001). Compared to survivors, nonsurvivors had significantly higher d-dimer (13.00 ± 3.20 mcg/mL vs 6.15 ± 3.66 mcg/mL; p< 0.006) and peak ferritin values (2763.66 ± 1105.32 ng/mL vs 835.16 ± 257.07 ng/mL; p= 0.016).

Conclusion: Our retrospective study shows COVID-19 infection may present as DKA in patients with diabetes mellitus (DM). Older age, mechanical ventilation, elevated d-dimer, and ferritin are associated with poor prognosis in these patients. Our study shows that COVID-19 is associated with substantial mortality in DKA patients and adds to the limited literature available regarding poor risk factors associated with mortality in these patients.

## Introduction

The severe acute respiratory syndrome-coronavirus-2 (SARS-CoV-2), an enveloped RNA beta-coronavirus, is a novel coronavirus responsible for the current global pandemic. It has led to a global health crisis with rapidly increasing number of cases and fatalities. The spectrum of clinical manifestations of COVID-19 infection includes fever, myalgia, cough and dyspnea, and less frequently headache, diarrhea, nausea, and vomiting [1]. Most infections are not severe. According to the Chinese Center for Disease Control and Prevention, 81% patients have mild-to-moderate disease (asymptomatic or mild pneumonia), 14% have severe disease (dyspnea, hypoxia, or >50% lung involvement on imaging within 24-48 h), and only 5% have critical disease with multiple organ failure (respiratory failure, shock, or multiorgan dysfunction) [2].

Studies have shown that the rate of diabetic ketoacidosis (DKA) admissions in the United States has increased by 59% from 2003 to 2014 although the mortality has decreased from 0.51% to 0.33% in the same timeframe [3]. Hence, DKA which was once a potentially fatal disease is now well manageable. With this study, we aim to highlight the risk factors which can predict increased mortality in patients being admitted with DKA and COVID-19 infection.

## Materials and methods

A retrospective analysis was conducted among patients admitted to our hospital in the United States between March 1st and June 15th, 2020 with DKA and SARS-CoV-2 infection known as COVID-19. COVID-19 was confirmed by real-time reverse-transcription polymerase chain reaction (PCR) assay. Diabetes was defined as glycated hemoglobin (HbA1c) ≥ 6.5% (48 mmol/mol) or any established diagnosis prior admission. DKA is defined by the triad of hyperglycemia (>250 mg/dL), anion gap metabolic acidosis, and ketonemia or ketonuria. The patients' electronic medical records were reviewed. Data on patients' age, sex, ethnicity, laboratory values, glycosylated hemoglobin level, oral antihyperglycemic agents (OHAs), insulin, and clinical outcomes were collected. 

## Results

We identified 43 patients admitted with COVID-19 and DKA from March 1^st^ to June 15^th^ of 2020. Pertinent clinical characteristics and laboratory values are summarized in Table 1.

**Table 1:** Pertinent clinical characteristics, laboratory values, and outcomes in our study population. IQR, interquartile range; BMI, body mass index; HbA1c, glycated hemoglobin; DM, diabetes mellitus; SGLT2, sodium-glucose co-transporter-2; DPPIV, dipeptidyl peptidase IV inhibitors; GLP-1, glucagon-like peptide-1; AKI, acute kidney injury; CRP, C-reactive protein; LDH, lactate dehydrogenase

Age (years)	
Median	52
IQR	40.5-64.5
Gender – no./total no. (%)	
Male	33/43(76.7)
Female	10/43(23.3)
Race – no./total no. (%)	
Hispanic	25 /43 (58.1)
Black	9/43(20.9)
White	7/43 (16.3)
Asian	1/43(2.3)
Arabic	1/43(2.3)
BMI (kg/m^2^)	
Median	26.7
IQR	24.2-29.9
Diabetes history – no./total no. (%)	
Type 1 DM	7/43(16.3)
Type 2 DM	26/43(60.5)
Previously undiagnosed diabetes	10/43(23.3)
Home medications – no./total no. (%)	
Insulin	19/43(44.2)
Oral hypoglycemic agents	
Metformin	21/43(48.8)
Sulfonyl urea	10/43(23.3)
SGLT2 inhibitors	6/43(14.0)
DPPIV inhibitors	4/43(9.4)
Thiazolidinediones	1/43(2.3)
GLP-1 agonist	1/43(2.3)
HbA1c on admission % (mmol/mol)- no./total no. (%)	
HbA1c ≥ 8% (≥ 64mmol/mol)	33/43(76.7)
HbA1c<8% (<64mmol/mol)	0/43(0.0)
HbA1c unknown	10/43(23.3)
Initial glucose value at presentation	
Median (range) – mg/L	553.0(300.0-1927.0)
Anion gap	
Median (range) – mEq/L	25.3(12.0-45.3)
D-Dimer	
Median (range) – mcg/mL	8.47(0.58-20)
Troponin	
Median (range) – ng/mL	0.1(0.01-39.0)
CRP	
Median (range) – mg/L	212.7(9.8-403)
LDH	
Median (range) – U/L	547.0(187.0-12000)
Ferritin	
Median (range) – ng/mL	1217.0(85.0-7500.0)
Lactic acid	
Median (range) – mmol/L	3.0(0.7-10.7)
Outcomes – no./total no. (%)	
Mechanical ventilation	22/43(51.2)
AKI	37/43(86.0)
Renal replacement therapy	6/43(14.0)
Death	25/43(58.1)

The median age for the patient was 52 years (inter quartile range, IQR 40.5-64.5). Thirty-three (76.7%) patients were male. Ethnicity distribution in our patients - 25 /43 (58.1 %) were Hispanic, 9/43 (20.9%) were African American, 7/43 (16.3 %) were White, 1 was Asian, and 1 was Arabic. Median body mass index (BMI) was 26.7 kg/m2. Out of 43 patients, 10 patients (23.3%) had undiagnosed diabetes mellitus (DM), seven patients (16.3%) had diagnosed type 1 DM and 26 patients (60.5%) had diagnosed type 2 DM. Amongst these 19 (44.2%) patients who were on insulin at home, 21 (48.8%) were on metformin, 10 (23.3%) were taking sulfonyl urea, 6 (14%) were on SGLT 2 inhibitors (known to increase the risk of ketoacidosis), 4 (9.3%) were on DPPIV inhibitors, 1 (2.3%) was on thiazolidinediones, and 1 (2.3%) was on GLP-1 agonist. On admission, 33 patients (76.7%) had HbA1c ≥ 8% (64 mmol/mol) and none had HbA1c < 8% (64 mmol/mol). HbA1c was not obtained in 10 (23.3%) patients. 

Out of 25 patients who died -- 15 (60.0%) were Hispanics, 6 (24.0%) were White, 3 (12.0%) were African American, and 1 was Arabic (4%). Median value of initial glucose on presentation in our study population was 553 mg/dL (300.0-1927.0 mg/dL). Median value of HbA1c on presentation was 13.9% (128 mmol/mol). Some 10 patients had large ketonemia, 17 patients had moderate ketonemia, and 16 patients had small ketonemia. Acute kidney injury (AKI) was seen in 37 patients (86.0%), 6 patients (14%) required renal replacement therapy, and 22 patients (51.2%) required mechanical ventilation. All the patients were started on standard treatment protocol for DKA with IV fluids and insulin infusion.

Table 2 shows analysis of factors associated with prognosis among patients with COVID-19 infection.

**Table 2:** Analysis of factors associated with prognosis among patients with COVID-19 infection. BMI, body mass index; AST, aspartate transaminase; ALT, alanine transaminase; CRP, C-reactive protein; LDH, lactate dehydrogenase; HbA1c, glycated hemoglobin; LDH, lactate dehydrogenase; ESR, erythrocyte sedimentation rate

Variable	Survivors	Nonsurvivors	p value
Age (years)	46 ± 9.39	58 ± 6.13	0.023
BMI (kg/m^2^)	26.97 ± 4.22	28.85 ± 2.52	0.4
HbA1c (%)	13.6 ± 1.19	13.37 ± 1.32	0.78
Glucose (mg/L)	673.44 ± 191.02	713.58 ± 157.05	0.735
Anion gap (mEq/L)	27.46 ± 3.79	25.92 ± 3.44	0.538
D-dimer (mcg/mL)	6.15 ± 3.66	13.00 ± 3.20	0.006
Troponin (ng/mL)	0.18 ± 0.17	3.76 ± 3.86	0.068
LDH (U/L)	601.16 ± 342.16	1289.39 ± 1023.84	0.197
AST (U/L)	78.72 ± 112.26	116.72 ± 96.58	0.598
ALT (U/L)	45.72 ± 46.06	61.08 ± 43.89	0.626
Lactic acid (mmol/L)	3.07 ± 1.06	3.856 ± 0.853	0.233
Fibrinogen (mg/dl)	555.66 ± 187.90	759.13 ± 181.26	0.121
Ferritin (ng/mL)	835.16 ± 257.07	2763.66 ± 1105.32	0.001
ESR	71.27 ± 20.49	66.48 ± 16.17	0.698
CRP (mg/L)	181.21 ± 61.61	229.46 ± 36.27	0.138

The patients who died were older than who survived (mean age 58 ± 6.13 vs 46 ± 9.39; p = 0.023). Some 95% of the patients requiring mechanical ventilation died (odds ratio [OR]: 89.25; 95% confidence interval [CI]: 9.103-874.9644); p = 0.001). Compared to survivors, nonsurvivors had significantly higher mean d-dimer (13.00 ± 3.20 mcg/mL vs 6.15 ± 3.66 mcg/mL; p < 0.006) and peak ferritin values (2763.66 ± 1105.32 ng/mL vs 835.16 ± 257.07 ng/mL; p = 0.016). There were no statistically significant differences between the two groups (survivors vs nonsurvivors) in BMI, HbA1c, glucose, anion gap, troponin, lactate dehydrogenase (LDH), aspartate transaminase, alanine transaminase, lactic acid, fibrinogen, male sex, AKI, and renal replacement therapy (all p > 0.05). 

## Discussion

In the United States, the prevalence of diabetes among adults varies with race/ethnicity and ranges from 6.8% to 15.3%. Diabetes is one of the leading causes of morbidity and mortality. More healthcare resources are estimated to be spent on diabetes than on any other condition [4]. Morbidity from diabetes is a consequence of both macrovascular disease (atherosclerosis) and microvascular disease (retinopathy, nephropathy, and neuropathy) [5]. The development and progression of these complications can be delayed with management of hyperglycemia.

The care of patients with diabetes during the COVID-19 pandemic poses unique challenges. COVID-19 can precipitate severe manifestations of diabetes, includingDKA and hyperosmolar hyperglycemic state (HHS) [6]. Kamrath et al. reported a significant increase in DKA at diabetes diagnosis during the COVID-19 period in 2020 as compared to the two previous years (44.7% in 2020 vs 24.5% in 2019; vs 24.1% in 2018) [7]. Guo et al. reported higher levels of inflammation-related biomarkers in DM patients compared to non-DM patients and DM as a risk factor for the progression and prognosis of COVID‐19 patients [8].

The exact pathological mechanism leading to the complication of DKA in COVID-19 is not known at present. Angiotensin-converting enzyme 2 (ACE2) serves as a functional receptor for SARS-CoV-2. ACE-2 is not only expressed in alveolar cells but also myocardial cells, proximal tubule cells of the kidney, ileum and esophagus epithelial cells, endocrine tissues of the pancreas, and bladder urothelial cells [9-10]. SARS-CoV-2 could cause direct tissue damage leading to acute hyperglycemia. 

The mortality rate in our study was much higher than that observed in the general population. Richardson et al. reported a mortality rate of 21% in a study of 5700 patients hospitalized with COVID-19 in the New York City area [11]. Another study demonstrated greater mortality in a subgroup of 24 patients with diabetes as compared to 26 patients without diabetes (16.5% vs 0%) [8]. Another study from New York, reported mortality of 50% in COVID-19 patients with DKA upon admission or developed during their hospital course [12]. In our study, we observed the same trend with higher mortality (58.1%) in COVID-19 patients with DKA. In contrary, another study showed that patients with DKA were more likely to survive compared to patients without DKA [13].

In our study, 51.2% of the patients required mechanical ventilation and 95% of these patients died (OR: 89.25; 95% CI: 9.103-874.9644; p = 0.01). Previously reported study has shown mortality of 88% in COVID-19 patients who were intubated [11]. However, they did not include the patients who were still alive in the ICU at the time of the study. Individuals of any age can acquire SARS-CoV-2 infection and older age has been associated with increased mortality [14-15]. In our study also, the mean age of nonsurvivors was 58 years while mean age of survivors was 46 years.

D-dimer was significantly higher on presentation in nonsurvivors vs survivors in our cohort. This is in concordance with the previous studies that have shown increased mortality in patients with elevated d-dimer [15-16]. Also, ferritin has been shown to be a marker of severity and poor prognosis in COVID-19 patients [17]. In our study the patients who died had a significantly higher peak ferritin level than those who did not. Male sex, obesity, and AKI have been previously associated with increased mortality; however, in our study these were not statistically significant (all p > 0.05) [14, 18-19]. Further studies with large sample size are needed. 

This study had some limitations. Firstly, our study is a single center retrospective observational study with no randomization between diabetic and nondiabetic patients and there are limitations to generalizing these findings. Secondly this study was a small number of COVID-19 patients with DKA in our sample. Thirdly, our center is a tertiary care center so most of the COVID-19 cases admitted had severe infection which could act as bias. Confounding by the use of steroids as part of COVID-19 treatment can also add to the limitations as the use of steroids can increase glucose levels. Confounding by comorbid conditions cannot be completely excluded.

## Conclusions

Our retrospective study shows COVID-19 infection may present as DKA in patients with DM. Older age, mechanical ventilation, elevated d-dimer, and ferritin are associated with poor prognosis in these patients. Our study shows that COVID-19 is associated with substantial mortality in DKA patients and adds to the limited literature available regarding poor risk factors associated with mortality in these patients. Further studies with large sample size are needed to further asses the clinical characteristics, prognostic factors, and outcomes in COVID-19 patients with DKA.
